# Activity in primary motor cortex during action observation covaries with subsequent behavioral changes in execution

**DOI:** 10.1002/brb3.550

**Published:** 2016-09-15

**Authors:** Nadav Aridan, Roy Mukamel

**Affiliations:** ^1^ School of Psychological SciencesTel‐Aviv UniversityTel‐AvivIsrael; ^2^ Sagol School of NeurosciencesTel‐Aviv UniversityTel‐AvivIsrael

**Keywords:** functional magnetic resonance imaging, imitation, mirror neurons, motor system

## Abstract

**Introduction:**

Observing someone else perform a movement facilitates motor planning, execution, and motor memory formation. Rate, an important feature in the execution of repeated movements, has been shown to vary following movement observation although the underlying neural mechanisms are unclear. In the current study, we examined how the rate of self‐paced index finger pressing is implicitly modified following passive observation of a similar action performed at a different rate.

**Methods:**

Fifty subjects performed a finger pressing sequence with their right hand at their own pace before and after passive observation of either a 1‐min video depicting the task performed at 3 Hz by someone else or a black screen. An additional set of 15 subjects performed the task in an MRI scanner.

**Results:**

Across all 50 subjects, the spontaneous execution rate prior to video observation had a bimodal distribution with modes around 2 and 4 Hz. Following video observation, the slower subjects performed the task at an increased rate. In the 15 subjects who performed the task in the MRI scanner, we found positive correlation between fMRI signal in the left primary motor strip during passive video observation and subsequent behavioral changes in task performance rate.

**Conclusion:**

We conclude that observing someone else perform an action at a higher rate implicitly increases the spontaneous rate of execution, and that this implicit induction is mediated by activity in the contralateral primary motor cortex.

## 1 Introduction

Passively observing actions performed by others influences subsequent actions performed by the observer. Such influences can take various forms including changes in reaction time, implicit or explicit imitation, and skill learning. For example, subjects are slower to respond to a visual cue if it depicts an action that is incongruent with the response—even though the content of the observed action is irrelevant to the task (Brass, Bekkering, & Prinz, 2001; Craighero, Bello, Fadiga, & Rizzolatti, 2002; Stürmer, Aschersleben, & Prinz, 2000). Action observation can also induce implicit changes in behavior such as a higher tendency to adopt the gestures and mannerisms of interacting partners (a phenomenon known as the Chameleon effect; Chartrand & Bargh, 1999; Ferguson & Bargh, 2004) or prime subsequent actions (Edwards, Humphreys, & Castiello, 2003). Furthermore, action observation has been shown to introduce gains in skill learning—even in the absence of physical practice (Cross, Kraemer, Hamilton, Kelley, & Grafton, 2009; Mattar & Gribble, 2005).

At the neural level, the mirror neuron system (MNS) has been suggested to support such phenomena. This system comprises neurons active during action execution that also respond during passive observation of similar actions (Rizzolatti & Craighero, 2004). Such neurons were originally described in the ventral premotor cortex of the macaque monkey (Gallese, Fadiga, Fogassi, & Rizzolatti, 1996; di Pellegrino, Fadiga, Fogassi, Gallese, & Rizzolatti, 1992) and later also in the parietal cortex (Fogassi et al., 2005; Rozzi, Ferrari, Bonini, Rizzolatti, & Fogassi, 2008) and motor cortices (Dushanova & Donoghue, 2010; Tkach, Reimer, & Hatsopoulos, 2007; Vigneswaran, Philipp, Lemon, & Kraskov, 2013). In humans, there is evidence for a network of regions with mirroring properties including the inferior parietal lobule, inferior frontal gyrus, ventral premotor cortex, and also regions that are less typically associated with the motor pathway (Gazzola & Keysers, 2009; Molenberghs, Cunnington, & Mattingley, 2012; Mukamel, Ekstrom, Kaplan, Iacoboni, & Fried, 2010). These neural circuits in humans have been suggested to be important for imitation, action understanding, and learning by observation (Iacoboni, 2009; Keysers, Kaas, & Gazzola, 2010; Rizzolatti & Sinigaglia, 2010). Indeed, observing an action for the explicit purpose of subsequent imitation evokes stronger activation in the MNS compared with observation for a different purpose such as visual discrimination (Buccino et al., 2004; Grèzes, Costes, & Decety, 1999; Suchan, Melde, Herzog, Hömberg, & Seitz, 2008).

The role of the MNS in explicit learning by observation has been examined in several imaging studies. Cross et al. (2009) report that observing a dance sequence that was trained—either physically or by observation—engages common regions within the left inferior parietal lobule and right premotor cortex. Importantly, observation of untrained dance sequences elicited lower responses in these regions. These results suggest a common substrate for observational and physical training in these regions. Few studies also examined the link between neural activity during action observation (training phase) and subsequent physical performance on the task. Frey and Gerry (2006) report a correlation between fMRI activity in the right intraparietal cortex during action observation with subsequent performance accuracy in a problem‐solving task. Along similar lines, Krüger et al. (2014) report correlation between fMRI activity during action observation in the right medial superior parietal lobule (SPL) and left parietal operculum with subsequent imitation accuracy. Other studies, using transcranial magnetic stimulation (TMS), point to a role of primary motor cortex (M1) in learning by observation (Avanzino et al., 2015; Brown, Wilson, & Gribble, 2009).

While these studies support a role of regions within the human MNS in explicit learning and imitation, much less is known about the role of the MNS in implicit imitation and whether its activation is automatic or requires explicit awareness. At the behavioral level it has been demonstrated that observing an action performed at a certain rate implicitly influences the spontaneous execution rate of a subsequent action performed by the observer (Bove et al., 2009). The aim of this study was to further explore this phenomenon and probe candidate brain regions involved in such implicit imitation of rate using whole‐brain fMRI. To this end, subjects performed a serial button‐pressing task at their own pace before and after observing a video of someone else performing the same task. We hypothesized that across subjects, activity levels in mirror neuron regions during action observation would correspond to the degree of subsequent implicit behavioral change.

## 2 Materials and Methods

### 2.1 Subjects

Fifty right‐handed healthy volunteers participated in the behavioral study (34 female, mean age 23.7 years, range 18–29 years) and another 15 right‐handed healthy volunteers participated in the fMRI study (10 female, mean age 25.0 years, range 22–30 years). All subjects had normal or corrected‐to‐normal vision, provided written informed consent to participate in the study, and were compensated for their time. The study was approved by the ethics committee at Tel‐Aviv University and Helsinki committee at Tel‐Aviv Sourasky Medical Center.

### 2.2 Behavioral study

#### 2.2.1 Procedure

**Figure 1:**
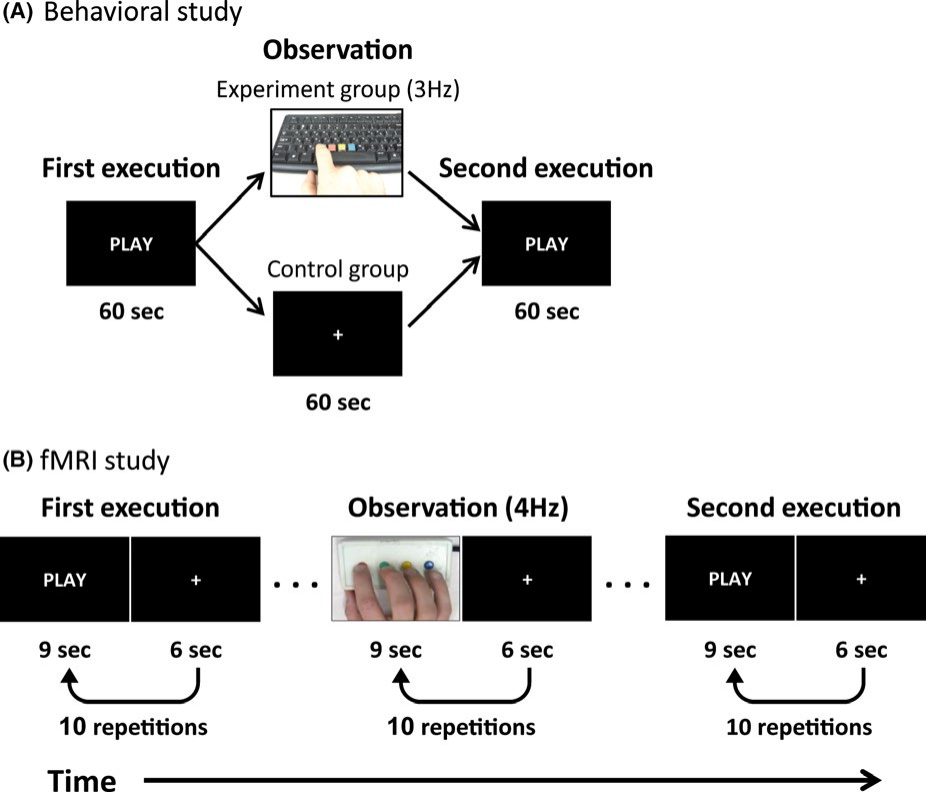
(A) Behavioral experiment design. Subjects performed a repeated serial button‐pressing task for 60 s at their own pace. This was followed by observation of a 60‐s video of someone else performing the task at a rate of 3 Hz (experiment group) or observation of a black screen (control group) for a similar duration. Finally, both groups performed the execution task a second time. (B) fMRI experiment design. Subjects performed 10 consecutive blocks of a repeated serial button‐pressing task at their own pace. Each 9‐s execution block was followed by 6 s of silent rest. The execution blocks were followed by 10 consecutive observation blocks of a video depicting someone else performing the task at a rate of 4 Hz. Finally, subjects performed the execution task again

Subjects performed a repeated serial button‐pressing task using their right index finger (execution task). They were instructed to sequentially press four color‐marked keys back and forth for 60 s at their own pace using their right index finger (sequence: 1‐2‐3‐4‐3‐2…). Each key produced a unique 90 ms duration note (E3, F3, G3, or A4) using “Midi‐ox” v7.0.2 software (http://www.midiox.com) and only one key could be pressed at a given time. Subjects performed the sequence for a few seconds before the beginning of the experiment in order to familiarize them with the task and to verify they understood it correctly. Following the initial execution task, subjects were randomly assigned to one of two groups. One group passively observed a 60‐s video of the same serial button‐pressing task performed by someone else at a rate of 3 Hz (“experiment group”; *N* = 25). Mean interpress interval (IPI) depicted in the video was 333 ± 11 ms. The other group observed a black screen (“control group”; *N* = 25) for the same time period. Subjects were instructed to fixate on the center of the screen and refrain from moving during the observation task. Finally, both groups performed the execution task again (Fig. 1A). Importantly, during action observation, subjects were not instructed to attend a particular feature of the video and participants in both groups did not know that they would be instructed to perform the execution task a second time. Individual subject performance was measured as the median of the interbutton press intervals (mIPI; in milliseconds) throughout each execution session separately. Changes in performance rate were calculated for each subject as the difference in this measure between the first and second execution tasks (ΔmIPI = mIPI_before − mIPI_after).

### 2.3 fMRI study

#### 2.3.1 Procedure

Subjects performed the serial button‐pressing task using their right hand (similar to the task described in the behavioral study procedure) while lying in an fMRI scanner (Fig. 1B). They were instructed to produce the same sequence of button presses as in the behavioral study (sequence: 1‐2‐3‐4‐3‐2…), back and forth at their own pace. As the subjects could not see their fingers, they used four different fingers (all digits except thumb), one finger for each key. Each key produced a different 70‐ms duration pure tone of 400, 500, 600, or 700 Hz and auditory feedback of the generated tones was provided via MR‐compatible “Optoacoustics” headphones (OptoActive). Only one key could be pressed at a given time. The subjects performed the sequence for a few seconds in the scanner before the beginning of the experiment in order to familiarize them with the task and verify they understood it correctly. Each 9 s experimental block was followed by 6 s of silent rest. Subjects performed 10 such execution blocks consecutively, followed by an observation task. In the observation task, subjects observed a video of someone else performing the same button‐pressing task at a rate of 4 Hz (10 repetitions of 9 s observation followed by 6 s of rest). Subjects were visually monitored to verify they did not move during the observation task. Finally, subjects performed the execution task again. The entire run lasted 7.5 min. As in the behavioral study, for each subject, the behavioral change in performance rate was calculated as the difference between the median interpress interval (mIPI; in milliseconds) before and after video observation (ΔmIPI = mIPI_before − mIPI_after).

#### 2.3.2 fMRI data acquisition and preprocessing

Functional imaging was performed on a 3T GE scanner with an 8‐channel head coil at the Sourasky Medical Center, Tel‐Aviv, Israel. For each subject, 39 interleaved ascending echo‐planar T2*‐weighted slices were acquired, providing whole‐brain coverage (slice thickness, 4 mm; slice gaps, 0 mm; in‐plane resolution, 1.72 × 1.72 × 4 mm; TR, 3,000 ms; TE, 35 ms; flip angle, 90°; field of view, 220 × 220 mm^2^; matrix size, 128 × 128). For anatomical reference, a whole‐brain high‐resolution T1‐weighted scan (voxel size, 1 × 1 × 1 mm) was acquired for each subject.

Functional magnetic resonance imaging data analysis was performed using “Brain Voyager QX” v. 2.8 software package (Brain Innovation, Maastricht, the Netherlands). Preprocessing of functional data included cubic spline slice time correction, trilinear/sinc three‐dimensional (3D) motion correction, temporal high‐pass filtering of 0.006 Hz, and spatial smoothing using a Gaussian filter (FWHM = 6 mm). Both anatomical and functional images were transformed into the standardized coordinate system of Talairach (Talairach & Tournoux, 1988). Experimental timeline was convolved with a standard hemodynamic response function (implemented in BVQX) and data analysis was performed using the general linear model (GLM).

In order to examine in which brain regions the activity patterns during passive action observation covary with the subsequent behavioral change in execution rate, we performed a whole‐brain regression analysis using the change in task execution rate for each participant (ΔmIPI = mIPI before observation − mIPI after observation), as a regressor against the activation levels of each voxel across all subjects during the observation session (β‐value of each subject during passive observation relative to baseline resting periods). The regression was corrected for multiple comparisons using *q*(FDR) < 0.05. In addition, we employed a more statistically lenient region of interest (ROI) approach specifically targeting regions of the MNS. Regions with mirroring properties were defined by performing a conjunction analysis targeting regions with significant activity during both the execution and observation tasks (first execution task > rest ∩ observation task > rest). To examine the correlation between the fMRI signal during action observation in these ROIs and subsequent changes in execution rate, we performed a regression analysis. We used the same behavioral measure as in the whole‐brain analysis as a regressor against the activation levels. The neural measure this time was the mean β‐value across all voxels in the ROI during the observation session relative to baseline. The regression was performed for each ROI separately.

## 3 Results

### 3.1 Behavioral study

**Figure 2:**
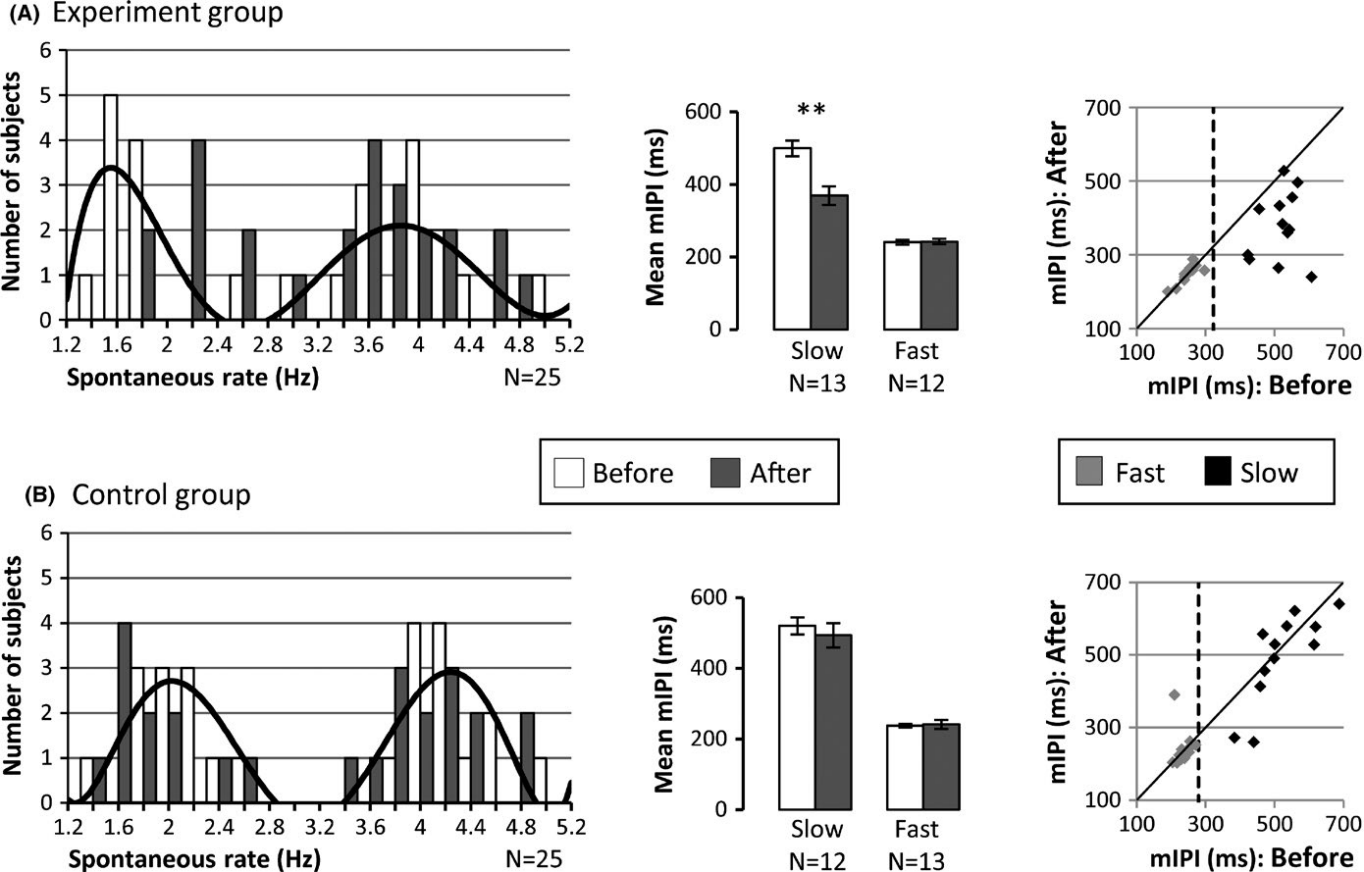
Behavioral results. (A) “Experiment group”: Spontaneous execution rate (left pane, white bars) had a bimodal distribution with modes of 1.8 and 4.2 Hz (labeled “slow” and “fast,” respectively) and median 3.1 Hz. The black line corresponds with a sixth order polynomial curve fit (*R* ^2^ = 0.46). In the slow subjects there was a significant difference between the rates (mean mIPI) of the first and second execution tasks (**p = 2.8 × 10^−4^), while in the fast subjects, there was no significant difference (group data are presented in the bar graph in the middle pane). Individual subject data are presented in the scatter plot on the right pane and the median spontaneous execution rate is marked by the dashed line. The black diagonal line represents identical mIPI across the two execution tasks. Dots below the line correspond with subjects that exhibited a faster (lower mIPI) rate in the second execution task. (B) Same as panel A for the “control group”: Spontaneous execution rate (left pane, white bars) had a bimodal distribution with modes of 2 and 4.1 Hz (“slow” and “fast,” respectively) with median 3.7 Hz (black line corresponds with a sixth order polynomial curve fit [*R* ^2^ = 0.64]). Both in the slow and fast subjects, there was no significant difference in rate (mean mIPI) between the first and second execution tasks

The distribution of spontaneous execution rates in each group was bimodal (modes were 1.6 and 4 Hz for the experiment group, and 2 and 4.1 Hz for the control group; see Fig. 2A and B, white bars). We therefore labeled subjects as “slow” or “fast”, according to their initial spontaneous rate (below or above the median: 3.1 Hz for the experiment group and 3.7 Hz for the control group) and examined them separately. Initial spontaneous rates were not significantly different between groups (“slow” experimental group vs. control and “fast” experimental vs. control). We found a significant three‐way interaction in the pressing rate between group type (slow/fast), observation condition (experiment/control), and time (before/after), *F*_1,46_ = 6.8, *p* < .05. Post hoc analysis (Tukey's HSD) showed that there was no significant difference in the initial spontaneous execution rate for the “slow” subjects who subsequently observed the 3 Hz video and “slow” subjects who observed the black screen (mean mIPI across subjects: 3 Hz video = 499 ms, black screen = 520 ms, *p* = .95). During the second execution session, subjects who observed the 3 Hz video performed the task at an increased rate (mean mIPI across subjects: before = 499 ms, after = 369 ms, *p* = 2.8 × 10^−4^). The group that observed a black screen did not exhibit a significant change in execution rate (mean mIPI across subjects: before = 520 ms, after = 493 ms, *p* = .73; Fig. 2). In the fast group of subjects there was no difference in the initial spontaneous performance rate between the group that subsequently observed the 3 Hz video and the group that subsequently observed a black screen (mean mIPI across subjects: 3 Hz video = 241 ms, black screen = 238 ms, *p* = .99). There was also no difference following the observation session (mean mIPI across the subjects who observed the 3 Hz video: before = 241 ms, after = 242 ms, *p* = .99; across the subjects who observed the black screen: before = 238 ms, after = 241 ms, *p* = .98; Fig. 2). Using the trough of the polynomial fit to separate the fast/slow subjects (instead of the median rate described above) yielded similar results (using this criterion resulted in one subject in the experimental group that switched label from slow to fast).

The results of the behavioral study demonstrate that when slow subjects observe an action performed at a rate that is faster than their initial execution rate, it induces an increase in their subsequent execution rate. Conversely, in the fast group, observing an action performed at a slower rate did not decrease the subsequent execution rate.

In order to understand the underlying neural mechanism of the behavioral results, we proceeded with an fMRI study. The spontaneous execution rate of the task across all 50 subjects (obtained from the first execution session) had a bimodal distribution with modes of 2 and 4.2 Hz, with a median of 3.5 Hz. Therefore, in the fMRI study, we decided to use videos depicting the button‐pressing task at a slightly higher rate of 4 Hz in order to enhance the behavioral effect in the smaller group of fMRI subjects.

### 3.2 fMRI study

The performance rate of the majority of fMRI subjects (12 of the 15) exhibited a spontaneous execution rate during the first session below 4 Hz (the rate displayed in the subsequent observation session). Repeated measures analysis of variance (ANOVA) of the performance rate in each block of the first execution session showed no significant difference in performance rate across the 10 blocks (*F*_1.8,25.7_ = 1.25, *p* = .3, Greenhouse–Geisser corrected) suggesting that the performance rate of the subjects was stable throughout the scan. Compatible with the results from the behavioral study, subjects’ performance rate increased following the observation session (mean mIPI across subjects: before = 390 ms, after = 296 ms, *p* < .002, one‐tailed paired *t* test).

**Figure 3:**
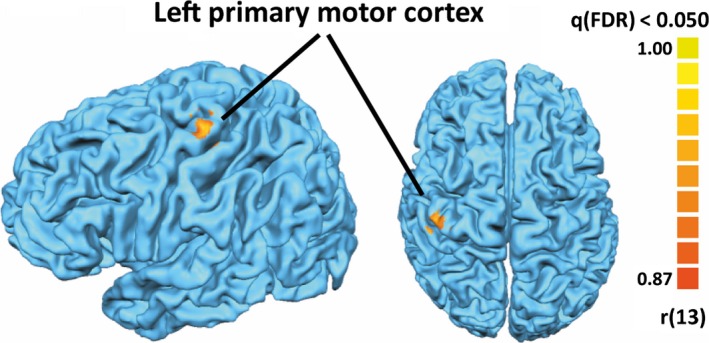
(A) Whole‐brain regression analysis (corrected using false discovery rate, *q*(FDR) < 0.05) revealed a significant positive correlation between the fMRI signal in the left primary motor cortex during video observation and the behavioral change in execution rate across subjects (ΔmIPI = mIPI before − mIPI after; mean Talairach coordinate: *x* = −42.5, *y* = −24.7, *z* = 67.2)

We performed a whole‐brain regression analysis in order to probe brain regions in which the fMRI signal during the passive observation session correlates with changes in task performance rate between the two execution sessions across all subjects (see Section “2”). We found a significant positive correlation in a single patch of voxels in the left primary motor cortex (273 significant voxels, whole‐brain RFX analysis corrected using false discovery rate, i.e., *q*(FDR) < 0.05; Fig. 3). Importantly, using the initial or postobservation execution rates as regressors (from the first or second execution tasks, respectively), as opposed to using the difference between the two execution tasks, yielded empty maps.

**Figure 4:**
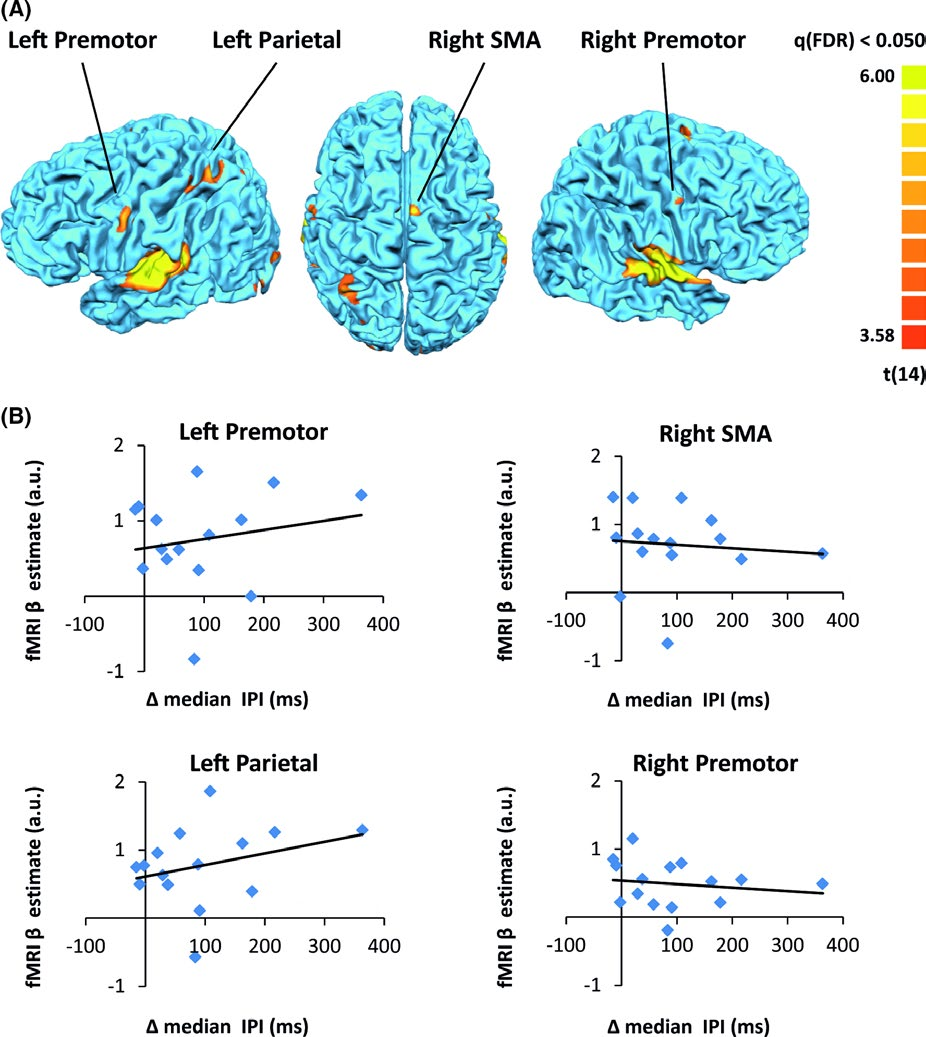
(A) Multisubject map (*N* = 15) showing ROIs within the mirror neuron system defined using a conjunction analysis of observation and execution (GLM contrast of first execution task > rest ∩ observation task > rest). The identified ROIs included the left and right premotor cortex, left SPL, and right SMA. (B) For each individual subject, fMRI β‐values of all voxels within an ROI were averaged and plotted against the behavioral change in execution rate (ΔmIPI = mIPI before − mIPI after). None of the ROIs exhibited a significant correlation (*r* values: left premotor cortex = 0.19, left SPL = 0.30, right SMA = −0.15, right premotor cortex = −0.09)

In addition, we functionally defined ROIs within the MNS by performing a conjunction analysis based on data obtained from the first execution task and the following observation task (first execution task > rest ∩ observation task > rest). Figure 4A displays the multisubject map of this contrast. The identified mirror ROIs included the following regions (number of voxels and Talairach coordinates): left premotor cortex (555 voxels, *x* = −57.4, *y* = −7.6, *z* = 40.6), left SPL (879 voxels, *x* = −34.3, *y* = −56.3, *z* = 58.1), right supplementary motor area (SMA; 244 voxels, *x* = 4.9, *y* = −7.0, *z* = 65.2), and the right premotor cortex (272 voxels, *x* = 54.0, *y* = −10.0, *z* = 45.8). Two additional clusters were found in the right and left superior temporal cortices, corresponding to auditory cortex, and are likely to be due to the auditory feedback which was present both in the execution and observation tasks. None of the ROIs showed a significant correlation between the mean β‐values across voxels obtained during the passive observation session and the subsequent change in task performance rate (ΔmIPI) across subjects.

## 4 Discussion

In the current study, we examined whether passive action observation, without the explicit purpose of future imitation, induces implicit behavioral changes in spontaneous movement rate. We find that observing an action indeed modulates subsequent performance rate, but only if the rate of the observed action is higher than the original execution rate of the observer. In our experimental design, subjects who observed an action performed at a lower rate did not show a decrease in their subsequent execution rate of the task. Our fMRI results point to the contralateral primary motor cortex as a candidate mediator of such implicit imitation.

Our behavioral study demonstrates that across subjects, spontaneous execution rate of the button‐pressing task has a bimodal distribution. We found that half of the subjects were centered on 2 Hz and the other half centered around 4 Hz. This is compatible with a previous study reporting a bimodal distribution of spontaneous tapping rate with similar frequency modes (Collyer, Broadbent, & Church, 1994). Other studies (Bove et al., 2009; McAuley, Jones, Holub, Johnston, & Miller, 2006; Vanneste, Pouthas, & Wearden, 2001) point to 2 Hz as a common natural spontaneous rate across subjects. It is possible that the additional 4 Hz frequency mode obtained from our subjects (and also in the study by Collyer et al., 1994) represents a harmony of the more commonly reported 2 Hz frequency. We note that our behavioral task was slightly different than the simple index finger tapping used in previous studies. Our behavioral task also included a spatial component as subjects were asked to press four different buttons back and forth with their index finger. Nonetheless, across the different variations in the task, it seems that 2 Hz is a common natural rate. Interestingly, it has been shown that a 3‐day physical training period can modulate this spontaneous rate in an attractor fashion—with training at a higher rate resulting in increased spontaneous rate and training at a lower rate resulting in decreased spontaneous rate in a subsequent test (Hammerbeck, Yousif, Greenwood, Rothwell, & Diedrichsen, 2014). In our short‐term experimental design (ten 9 s blocks in the fMRI study), we did not see significant within session learning effects.

Implicit induction of various movement parameters has also been demonstrated following passive observation of actions performed by others. For example, squeeze force (Obhi & Hogeveen, 2010) and grip force (Salama, Turner, & Edwards, 2011) have been shown to modulate according to the force of observed actions. Hand movement velocity is also implicitly influenced by the velocity of observed movement (Bisio, Stucchi, Jacono, Fadiga, & Pozzo, 2010), and the rate of a repetitive movement is another movement parameter that has been demonstrated to change following observation (Avanzino et al., 2015; Bove et al., 2009). In the case of movement rate, the study by Bove and colleagues reported higher execution rates in subject groups who observed a video depicting a high execution rate, and lower execution rates in subject groups who observed a video depicting a low execution rate (relative to the spontaneous rate of a control group who observed a neutral stimulus). In our pre/post within‐subject design, we found that using a video depicting an execution rate of 3 or 4 Hz (behavioral or fMRI studies, respectively) resulted in an increased execution rate in subjects who had a lower execution rate, but no decreased performance rate in those who were originally faster. It is an open question whether showing a very low performance rate (e.g., 1 Hz as used by Bove and colleagues) would have resulted in a decreased execution rate in the faster group. At least for the subjects with a spontaneous rate above 3 or 4 Hz in our study, showing an action performed at lower rate did not result in a reduction of their subsequent execution rate. The question why some subjects have a slower spontaneous tapping rate than others and why they are more susceptible to changes in spontaneous rate through visual induction deserves further study. Differences in spontaneous rate that lasted even up to 2 days following the initial observation session have been reported, suggesting that such effects are not temporally confined to the immediate timeframe following action observation (Bove et al., 2009). In the current study, the execution session immediately followed the observation session, thus our behavioral and fMRI results pertain to the immediate effects of action observation.

We examined the neural correlates of this implicit induction of execution rate using fMRI. Our whole‐brain analysis demonstrates that the level of activity in the left motor strip elicited during action observation correlates with subsequent changes in the execution rate across subjects. The initial or final execution rates alone (rather than the difference) did not correlate with fMRI activity. The subjects in both our experiments were visually monitored for movements, thus covert imitation during the observation task is unlikely to explain our result. We also specifically examined regions within the MNS as it has been implicated to play a functional role in automatic/implicit imitation. To this end, we defined the MNS by using a GLM conjunction analysis (execution > rest ∩ observation > rest). Adopting this, more statistically lenient ROI approach did not yield additional regions exhibiting a significant correlation with subsequent behavioral changes. However, this negative result should be taken with caution due to our limited sample size which may have resulted in low statistical power to detect such an effect in these regions.

Previous studies examined the relationship between neural activity during action observation, and subsequent performance level on a task similar to the one observed. Frey and Gerry (2006) had subjects observe a problem‐solving procedure and report that fMRI activity levels in the right intraparietal sulcus during observation correlated with subsequent performance accuracy. Using a bimanual imitation task, Krüger et al. (2014) report a positive correlation with behavior in the right SPL and the left parietal operculum and a negative correlation with behavior in the left IPL and the right vPMC. The abovementioned brain regions have been previously implicated in the MNS (Iacoboni et al., 1999; Molenberghs et al., 2012). It should be noted that both studies used a masked ROI analysis approach that did not include M1. Indeed, TMS stimulation of M1 has been shown to disrupt positive/negative effects of explicit learning by observation of congruent/incongruent actions, respectively (Brown et al., 2009). In our study, subjects were not instructed to attend any particular aspect of the observed stimulus and did not know that they would be asked to perform the execution task a second time. Furthermore, there was no element of performance level (whether explicit or implicit) in our task (i.e., performance‐wise, subjects had no particular incentive to tap at a particular rate). The changes in behavior we report are a result of implicit mimicry/contagion of the observed action and our whole‐brain fMRI results demonstrate that these implicit behavioral changes correlate with activity level in M1 during passive observation. This result is in agreement with our recent finding that visual presentation of actions that are not consciously perceived, is sufficient to elicit significant neural responses in frontal regions (Simon & Mukamel 2016). The current design does not allow determining which particular element of the observed action underlies these changes in behavior, however previous studies point to the importance of the presence of a biological agent in such effects (Avanzino et al., 2015; Kilner, Paulignan, & Blakemore, 2003).

Although the primary motor cortex (M1) is not classically considered an integral part of the core parietofrontal MNS (Iacoboni, 2005; Rizzolatti & Sinigaglia, 2010), there is accumulating evidence for M1 activity during action observation. Electrophysiological studies in monkeys demonstrated the existence of cells with mirroring properties in this region (Dushanova & Donoghue, 2010; Tkach et al., 2007; Vigneswaran et al., 2013; Waldert, Vigneswaran, Philipp, Lemon, & Kraskov, 2015). TMS studies further support the role of M1 in observational learning. Action observation has been shown to modulate TMS‐evoked excitability in M1 (Avanzino et al., 2015; Celnik et al., 2006; Stefan et al., 2005) and repetitive TMS to M1 has been shown to interfere with the behavioral effects of observational learning (Brown et al., 2009). Interestingly, the study by Vigneswaran et al. (2013) reports pyramidal tract neural activity in the primate primary motor cortex that is facilitated during action execution and suppressed during action observation. Along similar lines, a neuroimaging study in human reports increased fMRI BOLD signal during action execution and reduced signal during action observation (Gazzola & Keysers, 2009). Such lower activity levels in M1 during action observation might explain why this region is less consistently reported in the context of human mirroring studies (Caspers, Zilles, Laird, & Eickhoff, 2010).

The motor strip we detected using a whole‐brain regression analysis with behavior did not pass statistical threshold in our multisubject GLM observation/execution conjunction analysis. Therefore, it was not defined as part of the MNS network or examined in the ROI analysis. Closer inspection of this region revealed that this was mainly due to the greater signal variability across subjects during action observation. In some subjects, action observation elicited strong signals that passed the statistical threshold for a single‐subject GLM observation/execution conjunction analysis, while in others it did not. The fact that this variability across subjects correlated with their subsequent behavioral changes (as seen in the whole‐brain regression) suggests that this activity level in M1 during observation has functional significance. This variability across subjects might also explain why M1 is less frequently reported in imaging studies in the context of mirroring (when defined by observation tasks). Conversely, activation in the classical parietofrontal mirror neuron regions during action observation is more robust and consistent across subjects although, at least in our study, activity level in these regions did not correlate with subsequent implicit behavioral changes. Other studies using an explicit imitation/learning task report correlation with behavior within the parietofrontal MNS but their ROI masks did not include M1. Taken together, these studies suggest an interesting dissociation between the functional properties of mirroring activity across different regions within the MNS. An intriguing scheme integrating our results with the literature is one in which action observation elicits activity in the classical parietofrontal MNS, and the degree of its translation to behavior (at least during implicit imitation) depends on the relay of this information to M1. Subjects in which this relay is strong manifest higher levels of M1 activation during action observation and also stronger subsequent behavioral changes. The fact that our correlation with behavior during observation was found in M1 contralateral to the passively observed hand supports this view, although it deserves further study.

To conclude, we demonstrate that following exposure to a video depicting someone else perform a button‐pressing task, subjects tend to implicitly shift their spontaneous execution rate toward the higher rate of the observed action. The degree of this behavioral shift correlates with the degree of activation elicited during action observation in the contralateral motor cortex.

## Funding Information

This study was supported by the Israel Science Foundation (Grant/Award Number: 1771/13 and 2043/13), the Human Frontiers Science Project Organization (HFSPO) (Grant/Award Number: CDA00078/2011‐C), and the Israeli Centers for Research Excellence (Grant/Award Number: 51/11).

## Conflict of Interest

None declared.
